# SOX7-enforced expression promotes the expansion of adult blood progenitors and blocks B-cell development

**DOI:** 10.1098/rsob.160070

**Published:** 2016-07-13

**Authors:** Sara Cuvertino, Georges Lacaud, Valerie Kouskoff

**Affiliations:** 1 Stem Cell Hematopoiesis Group, Cancer Research UK Manchester Institute, University of Manchester, Wilmslow Road, Manchester M20 4BX, UK; 2 Stem Cell Biology Group, Cancer Research UK Manchester Institute, University of Manchester, Wilmslow Road, Manchester M20 4BX, UK

**Keywords:** SOX7, haematopoiesis, transcription factor

## Abstract

During embryogenesis, the three SOXF transcription factors, SOX7, SOX17 and SOX18, regulate the specification of the cardiovascular system and are also involved in the development of haematopoiesis. The ectopic expression of SOX17 in both embryonic and adult blood cells enhances self-renewal. Likewise, the enforced expression of SOX7 during embryonic development promotes the proliferation of early blood progenitors and blocks lineage commitment. However, whether SOX7 expression can also affect the self-renewal of adult blood progenitors has never been explored. In this study, we demonstrate using an inducible transgenic mouse model that the enforced expression of *Sox7 ex vivo* in bone marrow/stroma cell co-culture promotes the proliferation of blood progenitors which retain multi-lineage short-term engrafting capacity. Furthermore, SOX7 expression induces a profound block in the generation of B lymphocytes. Correspondingly, the ectopic expression of SOX7 *in vivo* results in dramatic alterations of the haematopoietic system, inducing the proliferation of blood progenitors in the bone marrow while blocking B lymphopoiesis. In addition, SOX7 expression induces extra-medullary haematopoiesis in the spleen and liver. Together, these data demonstrate that the uncontrolled expression of the transcription factor SOX7 in adult haematopoietic cells has dramatic consequences on blood homeostasis.

## 1. Introduction

The SOX family of transcription factors is highly conserved throughout evolution and in mouse comprises 20 members divided into subgroups based on sequence similarities [1]. During embryogenesis, SOX factors are essential for the regulation of many developmental processes, often implicating several SOX factors in one developmental path, such as, for example, SRY and SOX9 in sex determination or SOX1, 2 and 3 in neural tube formation [2]. In addition to their critical roles during embryogenesis, SOX factors are also often implicated in the maintenance and identity of stem cell populations [3]. For instance, SOX2 is critical for the expansion and maintenance of neuronal stem progenitor cells [4], while SOX9 determines mammary stem cell state [5].

SOX7 and its two close homologues, SOX17 and SOX18, belong to the SOXF subgroup, which are known to play important roles in cardiovascular development [6,7]. Compensatory mechanisms and some level of redundancy have been shown to occur between the three SOXF members during cardiovascular development and maintenance [8,9]. The SOXF factors have also been implicated in the development of the haematopoietic system, which is closely linked to endothelium development during embryogenesis [10]. SOX17 drives the expansion of fetal haematopoietic stem cells [11] while at the earliest stages of blood specification all three SOXF factors are expressed in haemogenic endothelium [12–15], a specialized subset of endothelium cells giving rise to blood progenitors upon endothelial to haematopoietic transition [16]. At E10.5 of embryonic development, the three SOXF factors are also found expressed in the endothelium lining of the dorsal aorta and in the emerging clusters of haematopoietic cells [8,17]. We and others have demonstrated that the ectopic expression of SOXF factors in haematopoietic cells leads to dramatic alterations in the balance between proliferation and differentiation. The enforced expression of SOX17 in adult blood cells was shown to enhance self-renewal capacity and to confer fetal-like haematopoietic properties; long-term ectopic expression of SOX17, however, led to leukaemogenesis [18]. Similarly, in E10.5 aorta-derived haematopoietic clusters, the enforced expression of SOXF factors, and SOX17 in particular, resulted in the maintenance and expansion of blood progenitors and HSCs [17]. A role for SOX17 in promoting the myeloid specification of E10.5 blood progenitors was also suggested in follow-up studies [19]. Our group showed that *Sox7* expression was upregulated in mesoderm precursors at the onset of blood specification and downregulated as differentiation progresses to committed blood lineages [13,14]. The enforced expression of SOX7 in E7.5-derived embryo cells or in *in vitro* differentiated embryonic stem cells was shown to promote the self-renewal of early blood progenitors harbouring endothelial-like features and to block further differentiation to committed lineages [13,14]. The enforced expression of SOX18 in these early embryonic populations led to a similar phenotype [15,17]. Given the potential of SOXF factors in maintaining the self-renewal properties of blood progenitors, we hypothesized that the ectopic expression of SOX7 may also confer a proliferative or survival advantage to adult haematopoietic cells. Using a transgenic inducible mouse model, we explore here the consequences of SOX7 ectopic expression on adult haematopoiesis both *ex vivo* and *in vivo*. We show that SOX7-enforced expression in adult bone marrow cells leads to the dramatic expansion of blood progenitors and specifically impairs B lymphopoiesis.

## 2. Material and methods

### 2.1. OP9 co-culture

*iSox7* bone marrow cells were plated on irradiated OP9 (30 cGy) in RPMI (Lonza) supplemented with 20% fetal calf serum (FCS), 5 µg ml^−1^ Kit ligand, 2 µg ml^−1^ Interleukin-7 and 5 µg ml^−1^ FLT3 (all PeproTech). When indicated, 1 µg ml^−1^ of doxycycline was added to the medium. Twice a week cells were harvested, counted and re-plated onto fresh irradiated OP9 cells.

### 2.2. Transplantation

Bone marrow cells were transplanted i.v. into sub-lethally irradiated (125 cGy) Nod Scid IL2rg-deficient mice (NSG, Charles River). After four weeks, mice were fed or not with doxycycline diet (Harlan). Mouse health was assessed by blood analysis, weight and general health monitoring.

### 2.3. Flow cytometry

Single-cell suspensions from adult bone marrow, spleen, liver and blood or OP9 co-culture were stained and analysed with FACSCalibur or LSRII and sorted with Influx or Aria flow cytometers (all BD Biosciences). Staining for sorting was performed in IMDM with 10% FCS, whereas cell surface staining for analysis was performed in PBS with 10% FCS. Cells were incubated with primary antibodies for 30 min at 4°C, then washed in PBS with 10% FCS and stained with secondary antibodies for 30 min at 4°C. After the secondary staining, cells were washed in PBS with 10% FCS and re-suspended in PBS with 10% FCS for cell surface staining or IMDM with 10% FCS for sorting. All antibodies and streptavidin used for staining were purchased from eBioscience. Details are available upon request. Data were analysed using the FlowJo software (TreeStar).

### 2.4. Clonogenic assay

Single-cell suspensions obtained from bone marrow, spleen or liver were plated at a density of 40 000 cells per dish in semi-solid medium supplemented with haematopoietic cytokines. The media contained 55% methylcellulose (10 g l^−1^), 10% serum (Stem Cell Technology), 10% protein-free hybridoma medium (PFM, Gibco), 2 mM l-Glutamine (Gibco), 180 µg ml^−1^ transferrin, 0.5 mM ascorbic acid, 4.5 × 10^−4^ M MTG, 1% Kit Ligand, 1% Interleukin-3, 1% thrombopoietin conditioned medium, 1 ng ml^−1^ Granulocyte–macrophage colony-stimulating factor, 5 ng ml^−1^ Interleukin-11, 2 U ml^−1^ Erythropoietin (Ortho-Biotech), 5 ng ml^−1^ Interleukin-6, 10 ng ml^−1^ macrophage colony-stimulating factor (M-CSF) (all from R&D system) and IMDM (Lonza). When indicated, 1 µg ml^−1^ of doxycycline was added to the semi-solid medium.

### 2.5. Immunohistochemistry

Reticulin staining was performed on paraffin sections using the Gordon and Sweet's stain. Sections were incubated for 5 min in a potassium permanganate (3% sulfuric acid) solution followed by washes in tap water. Next, 1.5% oxalic acid was applied until clear. After washes in tap water, sections were incubated with 2% ferric ammonium sulfate for 15 min. Multiple washes in distilled water were done before applying the ammoniacal silver solution (10% silver nitrate, ammonium hydroxide and 3% sodium hydroxide solution). Sections were washed in distilled water and fixed in 10% formalin for 5 min. After washes in tap water, slides were stained with 5% sodium thiosulfate for 2 min to remove unreduced silver, rinsed in tap water and incubated in 0.2% gold chloride for 3 min and then after washes in tap water, 0.1% neutral red was applied for 10 s. Finally, slides were dehydrated, cleared and mounted on clear glass covered by a coverslip.

For CD45R (B220) staining, sections were incubated with Dako target retrieval solution (S2369) at 125°C for 1 min, and then cooled to 90°C for 10 s. Slides were brought to room temperature in running water. Endogenous peroxidase was blocked in 0.3% TBS for 10 min. After washing thoroughly, blocking solution (10% rabbit serum) was applied for 30 min. Anti-CD45R antibody or Rat IgG2b control (BD Biosciences) was applied overnight at 4°C. After washing twice in TBS for 5 min, a rabbit anti-rat biotinylated antibody (Dako) was applied for 30 min at room temperature. Slides were washed twice in TBS and stained with Strep-AB-Complex–HRP (Dako). Sections were rinsed in running water and counterstained with 1X Gills Haematoxylin for 1 min, then washed in water for 1 min, dipped in alkaline water for 30 s and washed again in water for 1 min. Finally, slides were dehydrated, cleared and mounted on clear glass covered by a coverslip. Pictures were taken using a slide scanner machine (Leica SCN400).

### 2.6. May-Grünwald-Giemsa staining

Cells were cyto-spun onto slides, fixed with methanol for 10 min and stained with five volumes of o-dianidisine, one volume of 3% hydrogen peroxide and one volume of sodium nitroferricyanide mix for 10 min. After rinsing with water, slides were stained with May-Grünwald solution (Merck) for 3 min, rinsed with water and stained with a 1 in 20 dilution of Giemsa's stain solution (VWR) for 20 min. Slides were rinsed with water, left to dry and mounted with a coverslip. Haematoxylin and eosin staining was performed as previously described [20].

### 2.7. Transcriptomic analysis

Total RNA was extracted using the RNeasy Mini kit (Qiagen) and tested for RNA quality on an Agilent Bioanalyzer 2100 (Agilent Technologies). cDNA libraries were hybridized to Affymetrix mouse exon 1.0 ST array. Background adjustment and data normalization were performed with the robust multi-array average algorithm. Probesets were filtered by Affymetrix detection above background (DABG) scores. A probeset was kept if its DABG *p*-value was less than 0.01 in at least two samples. After these filtering steps, 189 956 probesets (representing 22 140 unique genes) from mouse array remained for differential gene expression analysis. Annotations and cross-mappings between probesets, exons and genes were obtained from Ensembl mouse genome using the R/BioConductor package Annmap (BioConductor 2.14).

### 2.8. Statistical analyses

Data were analysed using Student's *t*-test. Significant differences are indicates with *p < 0.05 and **p < 0.01.

## 3. Results

### 3.1. *Sox7*-enforced expression induces the proliferation of bone marrow progenitors and impairs B-cell differentiation

**Figure 1.**
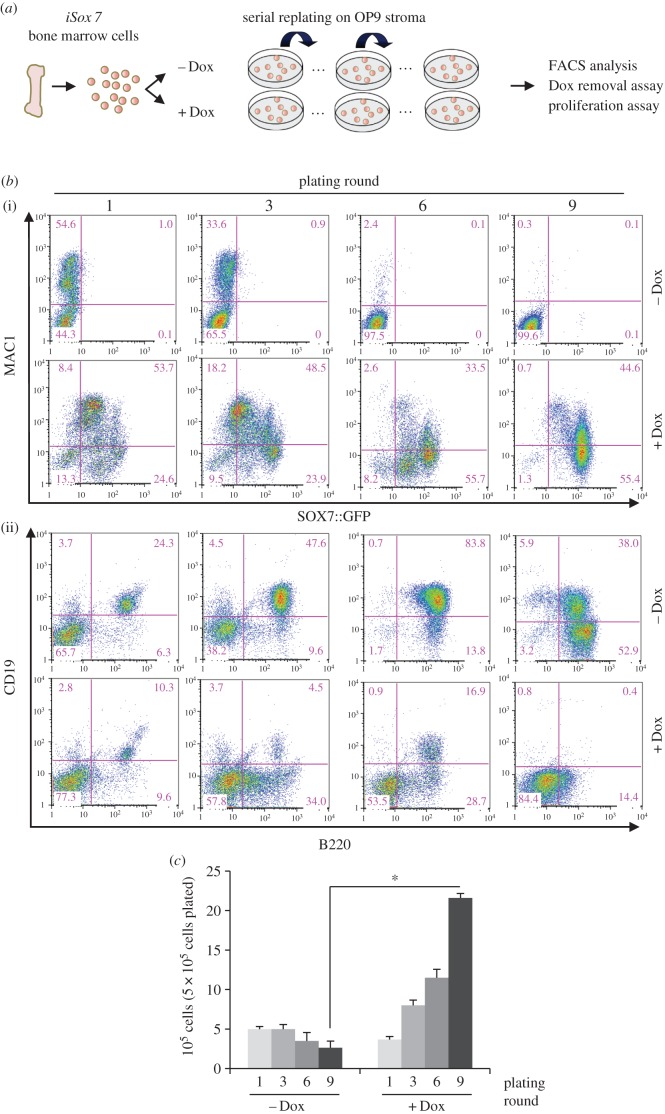
*Sox7*-enforced expression in bone marrow cells induces proliferation and impairs B-cell differentiation. (*a*) Schematic of the experimental strategy. (*b*) Flow cytometry dot plots showing the frequency of cells expressing (i) MAC1 and SOX7::GFP and (ii) B220 and CD19 from *iSox7* bone marrow-OP9 stroma cell co-cultured with (+Dox) and without doxycycline (−Dox). Data are representative of four independent experiments. (*c*) Absolute number of cells from *iSox7* bone marrow-OP9 stroma cell co-cultures serially passaged with or without doxycycline. Data are shown as the mean number of cells from three dishes; bars represent s.d. (*n* = 4, *p < 0.01).

**Figure 2.**
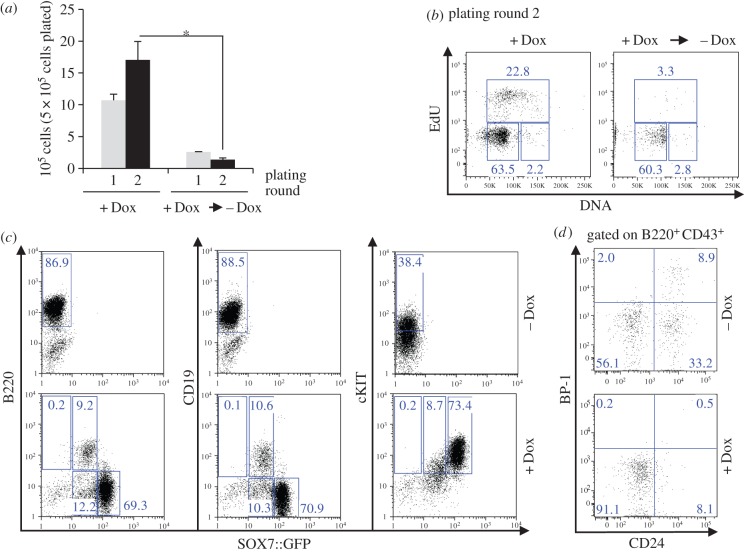
Maintenance of SOX7 expression is required for sustained proliferation. (*a*) After nine successive passages on OP9 stroma cells, *iSox7*^+^ bone marrow cells were cultured for two extra passages on OP9 with (+Dox) or without doxycycline (−Dox). Data are shown as the mean number of cells from three dishes; bars represent s.d. (*n* = 2, *p < 0.01). (*b*) Cell cycle status of *iSox7*^+^ bone marrow cells cultured with or without doxycycline (*n* = 2). (*c*) B220, CD19 and cKIT expression profiles of *iSox7* bone marrow cells cultured with or without doxycycline after nine successive passages (*n* = 4). (*d*) BP-1 and CD24 expression profiles of B220^+^CD43^+^ *iSox7* bone marrow cells cultured with or without doxycycline at plating round 9 (*n* = 4).

To determine whether SOX7-enforced expression in adult blood cells confers a proliferative advantage as observed during embryonic development, we investigated the consequences of *Sox7*-enforced expression on haematopoiesis. The impact of *Sox7* expression was first analysed using a murine bone marrow and OP9 stroma cell co-culture system allowing the close monitoring of haematopoietic differentiation to myeloid and lymphoid lineages [21]. To enforce *Sox7* expression, we used a previously described doxycycline-inducible transgenic mouse model for SOX7 and GFP co-expression (thereafter referred to as *iSox7*) [14]. Bone marrow cells were extracted from i*Sox7* mice and serially passaged on irradiated OP9 stroma cells with or without doxycycline (figure 1*a*). Without doxycycline, the frequency of MAC1^+^ myeloid cells progressively decreased while the frequency of B220^+^CD19^+^ B cells gradually increased, revealing the dynamic of this co-culture system, which gave rise to a wave of myelopoiesis followed by a wave of B lymphopoiesis (figure 1*b*). When *Sox7* expression was induced during the co-culture, the frequency of MAC1^+^ myeloid cells remained high upon successive passages with the progressive appearance of a MAC1^low^ population expressing SOX7::GFP at high level (figure 1*b*). By contrast, the generation of B220^+^CD19^+^ B cells was severely impaired and by nine passages no cells expressing B220 or CD19 were detected in the doxycycline-induced cultures. The total number of cells produced upon successive passages slowly decreased without doxycycline while a dramatic increase was observed upon *Sox7*-enforced expression (figure 1*c*). Most cells in the doxycycline-treated culture expressed CD45 and GFP (electronic supplementary material, figure S1*a*), and displayed increasing levels of *Sox7* expression upon serial passaging (electronic supplementary material, figure S1*b*). In addition, the *Sox7*-induced cultures contained more cells with an immature blast-like morphology and fewer B lymphocytes when compared with the control cultures (electronic supplementary material, figure S1*c*). We next examined whether *Sox7*-enforced expression was essential for the maintenance of the observed phenotype. After several passages, doxycycline was either removed or maintained in the culture and cell proliferation was compared between the two conditions. The expression of *Sox7* appeared critical for cell proliferation as the number of cells produced was dramatically and immediately reduced upon doxycycline removal (figure 2*a*). Cell cycle analysis revealed a massive decrease in the frequency of cells in the S-phase of the cell cycle upon doxycycline withdrawal (figure 2*b*).

We next investigated the immuno-phenotype of SOX7::GFP-expressing cells that accumulated over time in these cultures. Flow cytometric analysis revealed the presence of two subpopulations based on GFP expression level (figure 2*c*). A small SOX7::GFP^low^ population expressed B220 and CD19, and a predominant SOX7::GFP^high^ population did not express these B-cell markers but expressed high levels of cKIT. To further characterize the differentiation stage of the SOX7::GFP^low^B220^+^ population, a detailed analysis of early B-cell subsets was performed based on the expression of CD24 and BP-1, which divides the B220^+^CD43^+^ population into progenitor subsets [22]. All the subsets of developing B cells were observed in the control culture, while BP-1 and CD24 expression were not detected in the SOX7::GFP^low^ B220^+^CD43^+^ population, suggesting that these cells are blocked at a pre–pro B stage or earlier (figure 2*d*).

Taken together, these data revealed that the enforced expression of *Sox7 ex vivo* in adult bone marrow cells dramatically increases proliferation but impairs the differentiation of B lymphocytes.

### 3.2. The cKIT^+^SOX7::GFP^high^ subpopulation contains multi-lineage progenitors

**Figure 3.**
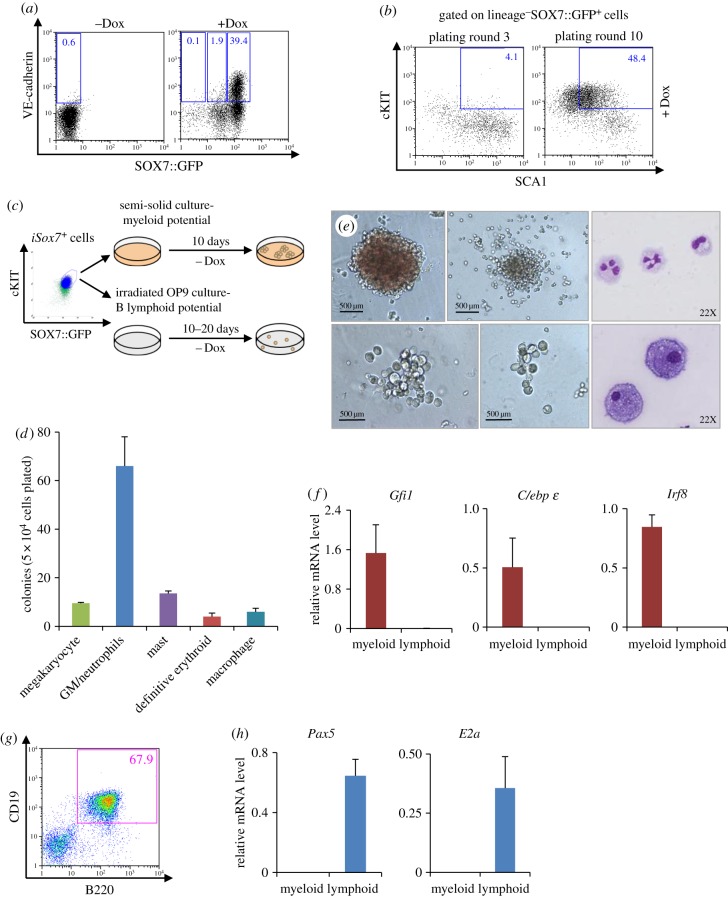
The cKIT^+^SOX7::GFP^high^ cell population contains multi-lineage progenitors. (*a*) VE-cadherin expression profiles of *iSox7* bone marrow cells cultured with (+Dox) or without doxycycline (−Dox) at plating round 9 (*n* = 4). (*b*) cKIT and SCA1 expression profiles of *iSox7*^+^ bone marrow cells gated on lineage^−^GFP^+^ at plating rounds 3 and 10 (*n* = 4). (*c*) Schematic of the experimental strategy. (*d*) cKIT^+^ SOX7::GFP^high^ cells from plating round 9 were sorted and replated in semi-solid clonogenic assay without doxycycline. Data shown are the mean numbers of colonies from three dishes; bars represent s.d. (*n* = 3). (*e*) Representative pictures of colonies and May-Grünwald-Giemsa staining of cells derived from colonies. Top panels (from left to right): erythroid, mast and granulocytes. Bottom panels (from left to right): macrophage, megakaryocyte and macrophages. (*f*) Q-PCR analysis for *Gfi1*, *C*/*ebpɛ* and *Irf8* transcript levels relative to *β*-*actin* in cells cultured in myeloid conditions without doxycycline. Data represent means ± s.d. (*n* = 3). (*g*) CD19 and B220 expression profile of cKIT^+^ SOX7::GFP^high^ cells cultured in lymphoid condition on OP9 stroma cells without doxycycline after 20 days in culture. (*h*) Q-PCR analysis of *Pax5* and *E2a* transcript levels relative to *β*-*actin* in cells cultured in lymphoid conditions without doxycycline. Data represent means ± s.d. (*n* = 3).

Over time, the frequency of the cKIT^+^SOX7::GFP^high^ subpopulation increased, suggesting that this subpopulation was specifically expanded by *Sox7* ectopic expression (electronic supplementary material, figure S2*a*). To define to which extent SOX7 expression altered this population, we investigated the immuno-phenotype and biological potential of these cKIT^+^ SOX7::GFP^high^ cells. The expression of VE-cadherin, a known transcriptional target of SOX7 [13], was observed on a large fraction of SOX7::GFP^high^ cells (figure 3*a*), further confirming SOX7 transcriptional activity in this population. The analysis of haematopoietic stem cell marker expression revealed a large increase in the frequency of the lineage^−^SCA1^+^cKIT^+^ fraction (figure 3*b*). To define the biological potential of this population, cKIT^+^SOX7::GFP^high^ cells were sorted and plated without doxycycline in clonogenic assay to test for myeloid and erythroid potential, and on OP9 co-culture to test for lymphoid potential (figure 3*c*). The cKIT^+^SOX7::GFP^high^ cells gave rise to erythroid as well as all types of myeloid colonies including megakaryocyte, granulocyte, mast and macrophage colonies (figure 3*d,e*); gene expression analysis for *Gfi1, C*/*ebpɛ* and *Irf8* further confirmed myeloid identity (figure 3*f*). When plated on OP9 stroma cells, cKIT^+^ SOX7::GFP^high^ cells generated B220^+^CD19^+^ B lymphocytes (figure 3*g*), with the expression of *E2a* and *Pax5* further confirming B-cell identity (figure 3*h*). To test for *in vivo* engrafting potential, sorted cKIT^+^SOX7::GFP^high^ cells were injected into immuno-compromised NSG recipient mice. After four weeks, peripheral blood cell analysis revealed the presence of CD45.2^+^ donor cells, indicating the success of the engraftment (electronic supplementary material, figure S2*b*–*c*). However, by 16 weeks, the frequency of CD45.2^+^ cells in the peripheral blood had dropped substantially, suggesting that only short-term engraftment capacity was maintained in the *ex vivo* derived cKIT^+^SOX7::GFP^high^ cell population.

Altogether, these results revealed that the enforced expression of *Sox7* in bone marrow cells leads to the *ex vivo* maintenance and expansion of a progenitor population able to give rise to myeloid, erythroid and lymphoid lineages and to provide short-term *in vivo* engraftment.

### 3.3. Molecular characterization of SOX7-mediated changes

**Figure 4.**
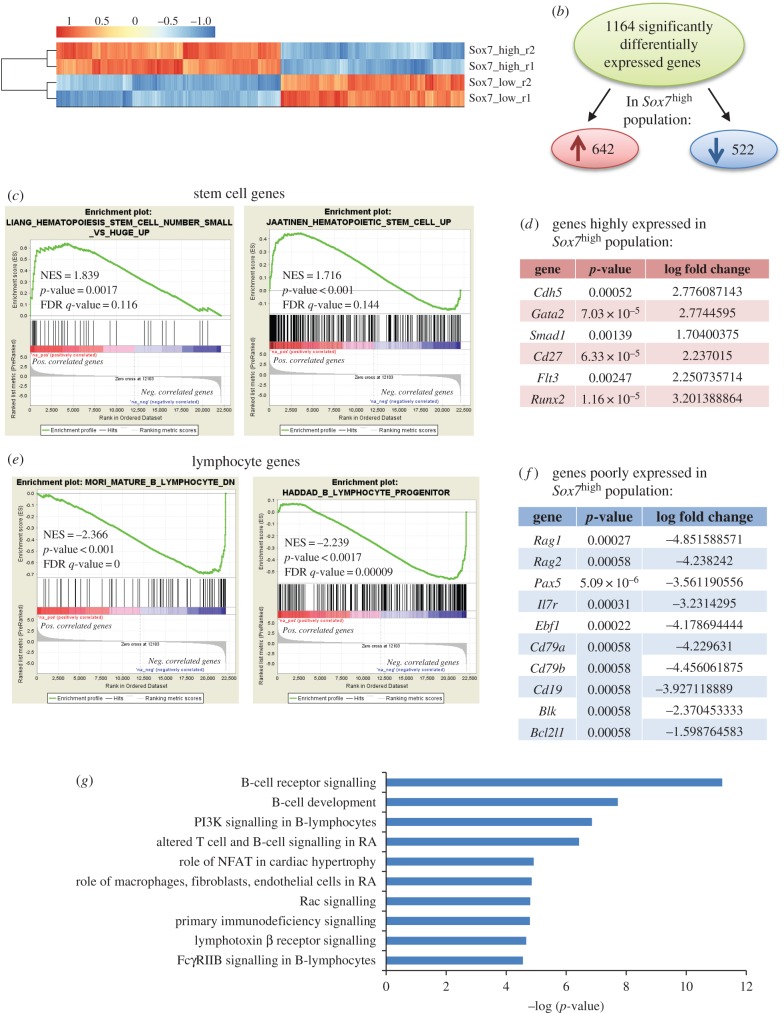
*Sox7*^high^ population is enriched for stem cell gene expression. (*a*) Heatmap of differentially expressed genes; expression levels changing by at least twofold between biological duplicates of SOX7::GFP^low^ and SOX7::GFP^high^ populations with a FDR < 5% were considered significant. (*b*) A total of 1164 genes were significantly differentially expressed. Of these, 642 genes were more highly expressed and 522 genes were less expressed in *Sox7*^high^ relative to *Sox7*^low^ populations. (*c*) GSEA demonstrates a significant enrichment for haematopoietic stem cell genes in the *Sox7*^high^ population. (*d*) List of some representative genes more expressed in *Sox7*^high^ relative to *Sox7*^low^ populations. (*f*) GSEA demonstrates a significant enrichment for B lymphocyte genes in the *Sox7*^low^ population. (*e*) List of some representative genes less expressed in *Sox7*^high^ relative to *Sox7*^low^ populations. (*g*) IPA canonical pathways analysis of genes lowly expressed in *Sox7*^high^ population (RA: rheumatoid arthritis).

To define at the molecular level the consequences of SOX7-mediated effect on proliferation and differentiation, transcriptome profiling analysis was performed on the two subpopulations expressing low and high levels of SOX7::GFP. Comparative analysis revealed that 1164 genes were significantly and differentially expressed, with 642 genes expressed at higher level and 522 genes expressed at lower level in SOX7::GFP^high^ cells relative to SOX7::GFP^low^ cells (figure 4*a,b*). Gene set enrichment analysis (GSEA) performed on all genes upregulated in the SOX7::GFP^high^ fraction demonstrated a significant enrichment for haematopoietic stem and progenitor cell genes [23,24] (figure 4*c*), an observation consistent with the haematopoietic multi-lineage potential of this population. Genes enriched on a high level of *Sox7* expression included stem/progenitor genes such as *Gata2* or *Flt3* (figure 4*d*). GSEA revealed a significant decrease in B lymphocyte-associated genes within the set of genes downregulated in the SOX7::GFP^high^ population [25,26] (figure 4*e*). The list of genes downregulated on *Sox7*-enforced expression included many B lymphocyte-associated genes such as *Rag1*/*2*, *Ebf1*, *Pax5* and *Cd19* (figure 4*f*). Canonical pathway analysis using the IPA software further revealed that most pathways involved in B-cell development and receptor signalling were downregulated by high expression of *Sox7* (figure 4*g*; electronic supplementary material, figure S3).

Taken together, these data clearly establish the profound impact of *Sox7* expression on the normal differentiation of B cells at the molecular level and further validate the flow cytometry data, suggesting that the enforced expression of *Sox7* in adult bone marrow cells impairs B-cell maturation, while promoting a stem/progenitor signature.

### 3.4. *Sox7*-enforced expression *in vivo* disrupts the homeostasis of the haematopoietic system

**Figure 5.**
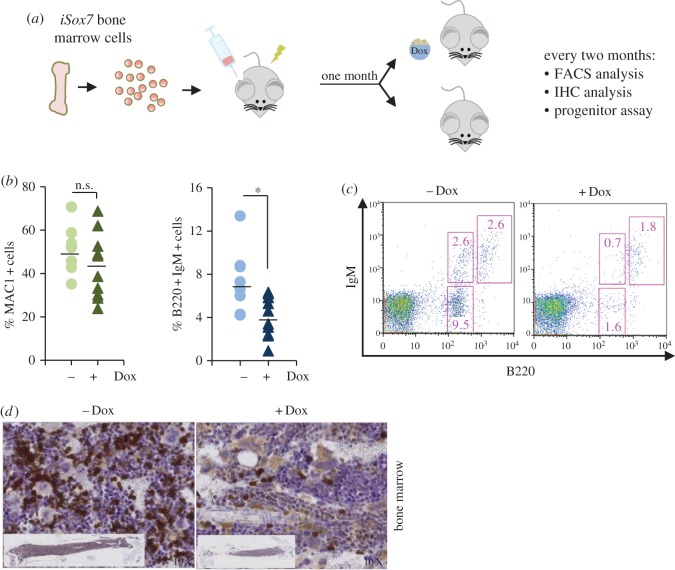
*Sox7*-enforced expression impairs B-cell differentiation *in vivo*. (*a*) Schematic of the experimental strategy. (*b*) MAC1^+^ and B220^+^IgM^+^ cell frequencies in the bone marrow of animal fed (+Dox) or not (−Dox) with doxycycline. Each dot represents an individual mouse. Bars represent the mean percentage from all mice analysed between 2 and 12 months (*p < 0.05). (*c*) B220 and IgM expression profiles of bone marrow cells extracted from mice fed or not doxycycline at 11 months. (*d*) Immunohistochemistry of femur sections stained for B220 expression and counter stained with May-Grünwald-Giemsa.

**Figure 6.**
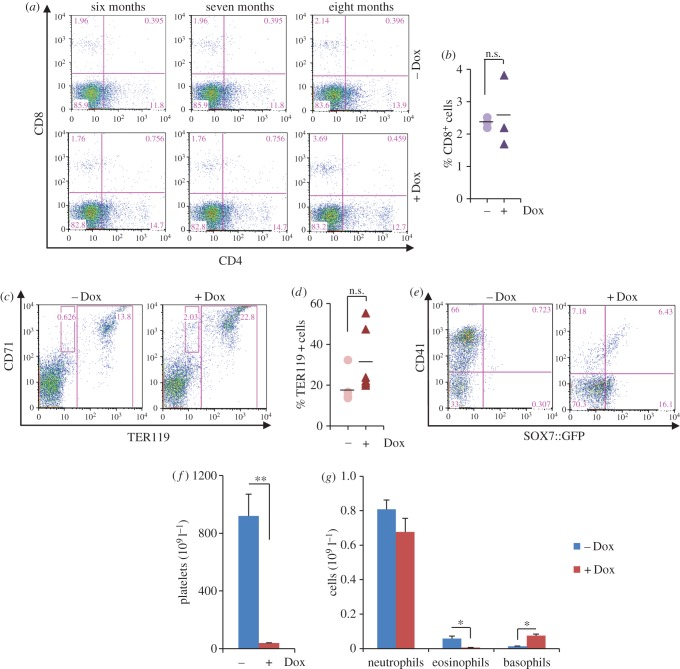
Multi-lineage analysis of the consequences of *Sox7*-enforced expression. (*a*,*c*,*e*) Representative FACS plots of the expression profiles in bone marrow cells for (*a*) CD8 and CD4, (*c*) TER119 and CD71, and (*e*) CD41 and SOX7::GFP (gated on large cells) from mice fed with or without doxycycline (dox). (*b*,*d*) Scatter charts presenting the percentages of (*b*) CD8^+^ cells and (*d*) TER119^+^ cells in the bone marrow of mice fed or not with doxycycline. Bars indicate mean, each dot represents an individual mouse (n.s.: non-significant). (*f*) The bar chart shows platelets count in the blood of mice fed with or without doxycycline. Error bars indicate mean ± s.d. (*n* = 6, **p < 0.005). (*g*) The bar chart shows neutrophil, eosinophil and basophil counts in the blood of mice fed with or without doxycycline. Error bars indicate mean ± s.d. (*n* = 6, *p < 0.05). All graphs are representative of two independent experiments.

Given the impact of *Sox7* expression in ex *vivo* cultures, we next investigated whether *Sox7*-enforced expression *in vivo* affects the homeostasis of the haematopoietic system. First, recipient mice were engrafted with *iSox7* bone marrow cells to establish a model system in which *Sox7* expression is only inducible in the haematopoietic system. One month post-engraftment, mice were then fed or not with doxycycline diet and monitored over a 12-month period (figure 5*a*). *Sox7*-enforced expression *in vivo* resulted in a progressive increase in the frequency of SOX7::GFP^+^ cells in blood, bone marrow, spleen and liver (electronic supplementary material, figure S4*a*). *Sox7*-enforced expression did not affect the frequency of MAC1^+^ cells in the bone marrow, while the frequency of B220^+^IgM^+^ cells was significantly decreased (figure 5*b*), with a strong reduction in the frequency of both mature and immature populations (figure 5*c*). Immunohistochemistry analysis confirmed the dramatic decrease in B220-expressing cells in the bone marrow upon *Sox7*-enforced expression (figure 5*d*), while collagen staining revealed important fibrosis in the bone marrow (electronic supplementary material, figure S4*b*). In contrast to B cells, the frequencies of CD4^+^ and CD8^+^ T cells (figure 6*a,b*) as well as TER119^+^ erythrocytes (figure 6*c*,*d*) were not significantly affected by *Sox7*-enforced expression in the bone marrow of animals fed with doxycycline when compared with control animals. However, we did observe a sharp decrease in CD41^+^ megakaryocytes in the bone marrow of the treated animal (figure 6*e*) that was reflected by a dramatic decrease in platelet count in the blood of these animals (figure 6*f*). Finally, while neutrophil count was not significantly affected by *Sox7*-enforced expression, eosinophil count was decreased and basophil count was increased in the blood of the treated animals (figure 6*g*). It is not clear, however, if these changes were a direct consequence of SOX7-enforced expression or if they represented secondary effects due to fibrosis in the bone marrow.

**Figure 7.**
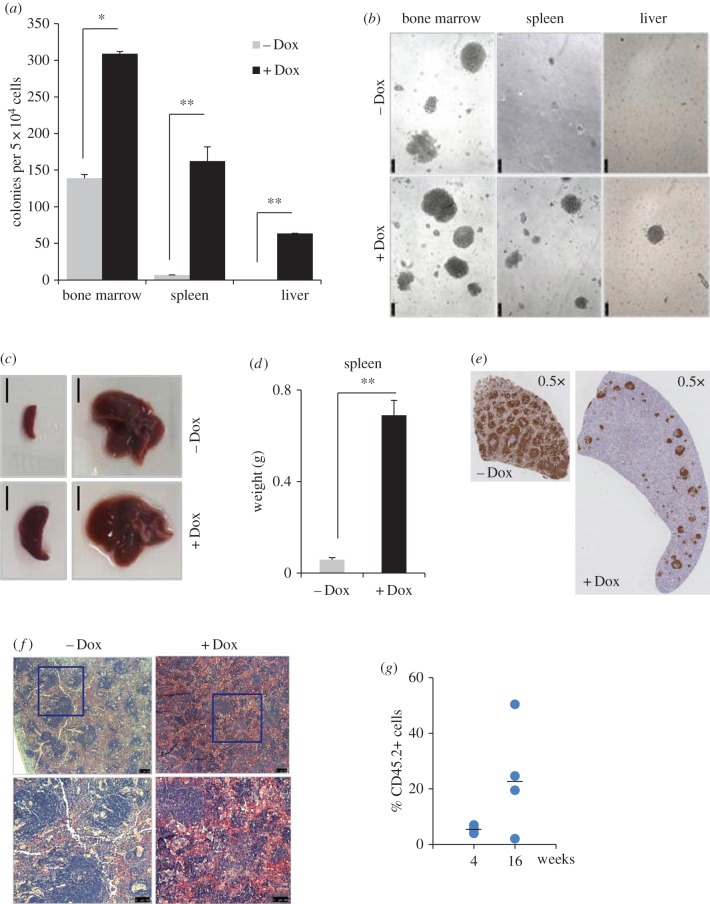
SOX7 ectopic expression induces extra-medullary haematopoiesis. (*a*) Bone marrow, spleen and liver cells from engrafted mice fed (+Dox) or not (−Dox) with doxycycline were cultured in clonogenic assays without doxycycline. Data represent mean number of colonies from three dishes, bars represent s.d. (*n* = 3, **p < 0.01, *p < 0.05). (*b*) Representative pictures of colonies obtained in the clonogenic assays (scale bar, 100 µm). (*c*) Representative pictures of spleen and liver of mice fed or not with doxycycline diet (scale bar, 0.75 cm). (*d*) Data showing spleen weight in grams, bars represent s.d. (*n* = 8, ** *p* ≤ 0.01). (*e*) Immunohistochemistry of spleen sections stained for B220 expression and counter-stained with May-Grünwald-Giemsa. (*f*) Haematoxylin-and-eosin-stained spleen sections of mice engrafted with *iSox7* bone marrow cells fed or not with doxycycline diet (scale bar, 100 µm), higher magnification in the bottom panels. (*g*) CD45.2^+^ cell frequency in the peripheral blood of mice transplanted with spleen cells of primary mouse fed with doxycycline diet; each dot represents an individual mouse, and bars represent mean frequencies.

We next investigated whether *Sox7*-enforced expression also affected haematopoietic progenitors normally residing in the bone marrow. Upon clonogenic replating, the bone marrow cells of doxycycline-fed animals gave rise to two times more colonies than the control bone marrow cells (figure 7*a*,*b*). Altogether, these findings demonstrate that *in vivo* the ectopic expression of SOX7 leads to B lymphopoiesis impairment and expansion of haematopoietic progenitors in the bone marrow associated with fibrosis.

The induced expression of *Sox7* also led to splenomegaly and partial hepatomegaly (figure 7*c*,*d*), associated with a dramatic decrease in B220-expressing cells (figure 7*e*) and fibrosis (electronic supplementary material, figure S4*b*). The two main compartments in the spleen, white pulp and red pulp, were profoundly disorganized (figure 7*f*). The red pulp is involved in filtering the blood and recycling iron from old erythrocytes, while the white pulp represents the lymphoid regions within the spleen. Upon SOX7-enforced expression, the lymphoid follicles were disrupted compared with the control samples, where a clear distinction between the follicles and red pulp was visible. The red pulp did not seem to be overtly affected by SOX7 expression. Disruption of the spleen architecture is probably due to the loss of B cells in the *Sox7*-expressing samples.

We next investigated the presence of extra-medullary haematopoiesis in these two organs. Clonogenic assays demonstrated a dramatic increase in the frequency of haematopoietic progenitors in both spleen and liver (figure 7*a*,*b*). To investigate the presence of multi-potent engrafting progenitors in extra-medullary haematopoietic sites, recipient mice were engrafted with spleen cells derived from doxycycline-fed animals. Analysis at 4 and 16 weeks post-engraftment revealed the presence of CD45.2^+^ donor-derived cells in the peripheral blood of recipient mice (figure 7*g*). At 20 weeks post-engraftment, contribution of CD45.2^+^ cells to erythroid, myeloid and lymphoid lineages was observed in the spleen of the recipient mice (electronic supplementary material, figure S4*c*).

Together, these data reveal that *Sox7*-enforced expression *in vivo* impairs B lymphopoiesis and expands the haematopoietic progenitor compartment, resulting in splenomegaly and extra-medullary haematopoiesis.

## 4. Discussion

Using an inducible transgenic mouse model system, we demonstrate here that the ectopic expression of SOX7 in adult bone marrow cells promotes the self-renewal of immature blood progenitors, leads to a profound block in B-cell development and globally perturbs the homeostasis of the haematopoietic system with extra-medullary haematopoiesis and extensive fibrosis in spleen and bone marrow.

The findings presented here clearly demonstrate that the ectopic expression of SOX7 has dramatic consequences for the balance between self-renewal and differentiation of blood progenitors. This is very similar to what has been observed for SOX17, for which ectopic expression was shown to alter both embryonic [17] and adult haematopoiesis [18]. Likewise, SOX18 ectopic expression was shown to promote the self-renewal of embryonic blood progenitors [15,17]. It is currently unknown whether SOX18 mis-expression in adult blood cells might also affect the balance between self-renewal and lineage commitment. The three SOXF factors have been shown to compensate for each other on mixed genetic background [8,9], however, when backcrossed to homogeneous background, for reasons that are still unclear, these compensatory mechanisms are lost. Additional studies have revealed a specific function for SOX18 in lymphangiogenesis [27] and various functions for SOX17 in endoderm specification [28], HSC proliferation [18] or arterial-venous specification [29] according to the cellular compartment in which this factor was deleted. To date, the specific role of SOX7 remains unknown, but SOX7 was shown to be expressed in primitive endoderm [30] and in vasculature [8]. Its deletion is linked to diaphragmatic hernia, cardiac oedema and apparent lack of yolk sac vasculature [31], but further characterization of SOX7-deficient embryos will be required to fully understand the main function of SOX7 during embryonic development.

When ectopically expressed in blood progenitors, all SOXF factors are able to alter the balance between proliferation and differentiation. Given their known degree of redundancy, it is very likely that all three SOXF factors activate a similar transcriptional programme that promotes proliferation at the expense of lineage differentiation. Based on the known functions of the SOXF factors, two possible explanations can be suggested for these observations: (i) the transcriptional programme activated by all SOXF in blood cells is similar to the one activated by SOX17 in fetal HSCs, which promotes the active proliferation of these cells [11]; SOX7 and SOX18 are able to activate this programme when ectopically expressed in a blood-specific cell context. (ii) All three SOXF factors are expressed in haemogenic endothelium [12,13] and might in this cellular context induce a transcriptional programme promoting proliferation which can be induced in a blood-specific cell context upon ectopic expression. The transcriptional targets specifically activated by the SOXF factors to regulate self-renewal are still currently unknown; this will be an important avenue of investigation for future studies.

Since the ectopic expression of all three SOXF factors induced such a dramatic shift from differentiation to self-renewal, it is surprising that the expression of these factors is not found misregulated in leukaemias. While the long-term ectopic expression of SOX17 ultimately leads to leukaemogenesis in mouse models [18], this factor has never been implicated in human haematological malignancies. Similarly, mutations in SOX18 have been linked to hypotrichosis–lymphedema–telangiectasia [32] but to date no change in SOX18 expression has been associated with blood disorders. By contrast, a role for SOX7 was recently described in acute myeloid leukaemia (AML), where this transcription factor was shown to block proliferation [33], acting in that context as a tumour suppressor. This role for SOX7 in AML is in striking contrast to the data presented here and to the block induced by SOX7-enforced expression in B lymphopoiesis. This difference raises the possibility that in normal haematopoietic development SOX7 might be implicated in the commitment or proliferation of progenitors to B-lymphocytes versus myeloid lineages. During embryonic development, progenitor populations restricted to B-cell/macrophages [34] or B-cell/T-cell/macrophages [35] have been described. Given the known expression of SOX7 at the onset of blood development in the embryo [13], it is possible that SOX7 is part of the transcriptional machinery promoting the proliferation and/or commitment of these progenitors towards B cells or macrophages. The tumour suppressive role of SOX7 in AML was directly linked to the inactivation of the Wnt/β catenin pathway [33]. However, β catenin has been shown to be dispensable for B-cell development [36], suggesting that the mode of action of SOX7 in myeloid and lymphoid lineages is likely to be quite different. It will be important in future studies to elucidate how SOX7 might exert opposite effects in myeloid and lymphoid lineages.

## Supplementary Material

Supplementary figures

## Data accessibility

The microarray data reported in this study are accessible via the NCBI Gene Expression Omnibus webpage and the accession number is GEO: GSE65552.
