# Supplementary material for: SOX7-enforced expression promotes the expansion of adult blood progenitors and blocks B-cell development
Source: Open Biol. 2016 Jul 13;6(7):160070. doi: 10.1098/rsob.160070 (PMC4967825; doi:10.1098/rsob.160070)
Supplement: Supplementary figures [file rsob160070supp1.pptx]

## Slide 1
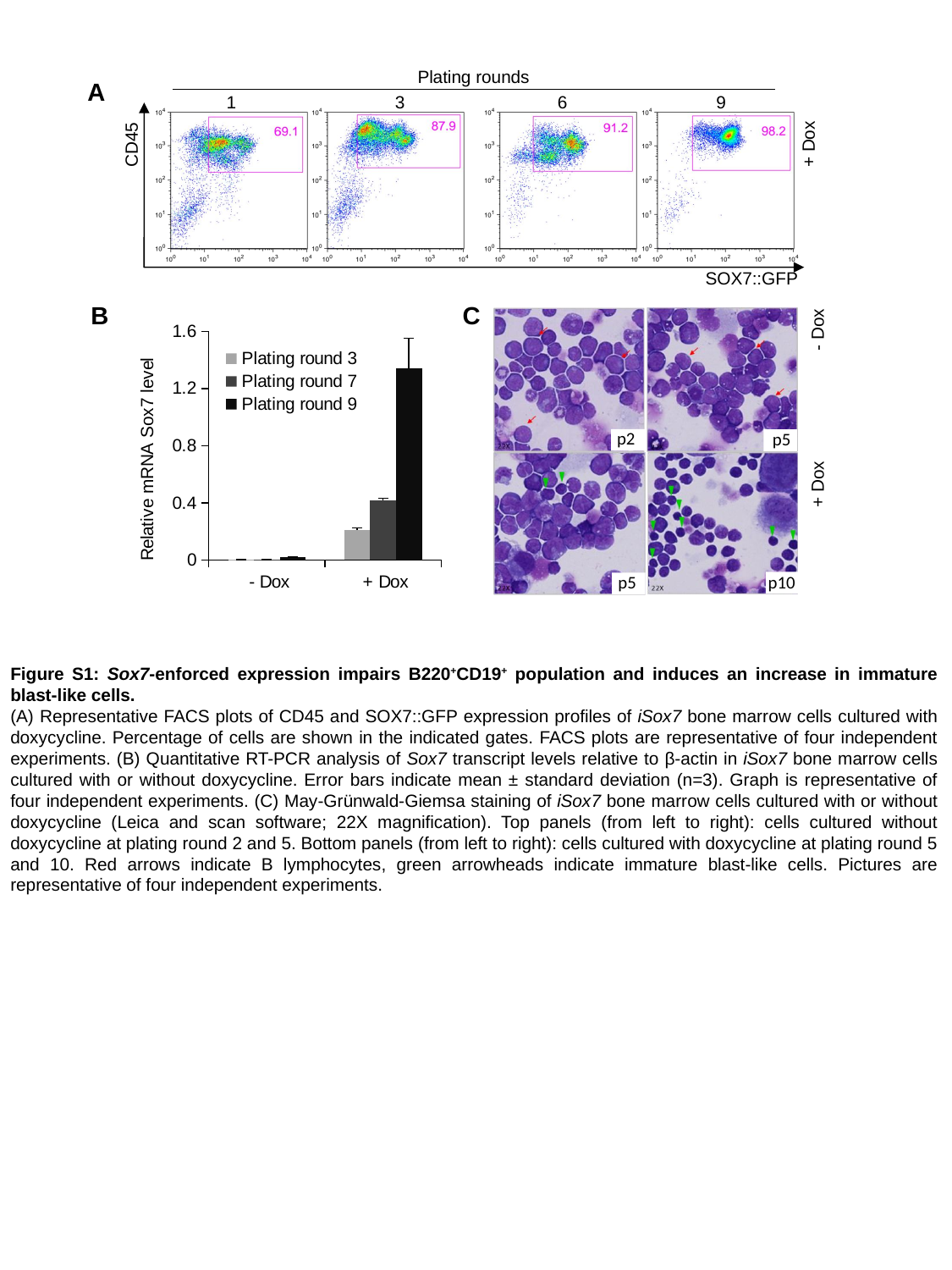

Plating rounds
A
1
3
6
9
+ Dox
CD45
SOX7::GFP
B
C
- Dox
### Chart
| Category | | | |
|---|---|---|---|
| - Dox | 0.00238533881718092 | 0.00036502530133907 | 0.0170832310941663 |
| + Dox | 0.211676484104023 | 0.416280455961179 | 1.343880100099843 |p2
p5
+ Dox
p10
p5
Figure S1: Sox7-enforced expression impairs B220+CD19+ population and induces an increase in immature blast-like cells.
(A) Representative FACS plots of CD45 and SOX7::GFP expression profiles of iSox7 bone marrow cells cultured with doxycycline. Percentage of cells are shown in the indicated gates. FACS plots are representative of four independent experiments. (B) Quantitative RT-PCR analysis of Sox7 transcript levels relative to β-actin in iSox7 bone marrow cells cultured with or without doxycycline. Error bars indicate mean ± standard deviation (n=3). Graph is representative of four independent experiments. (C) May-Grünwald-Giemsa staining of iSox7 bone marrow cells cultured with or without doxycycline (Leica and scan software; 22X magnification). Top panels (from left to right): cells cultured without doxycycline at plating round 2 and 5. Bottom panels (from left to right): cells cultured with doxycycline at plating round 5 and 10. Red arrows indicate B lymphocytes, green arrowheads indicate immature blast-like cells. Pictures are representative of four independent experiments.

## Slide 2
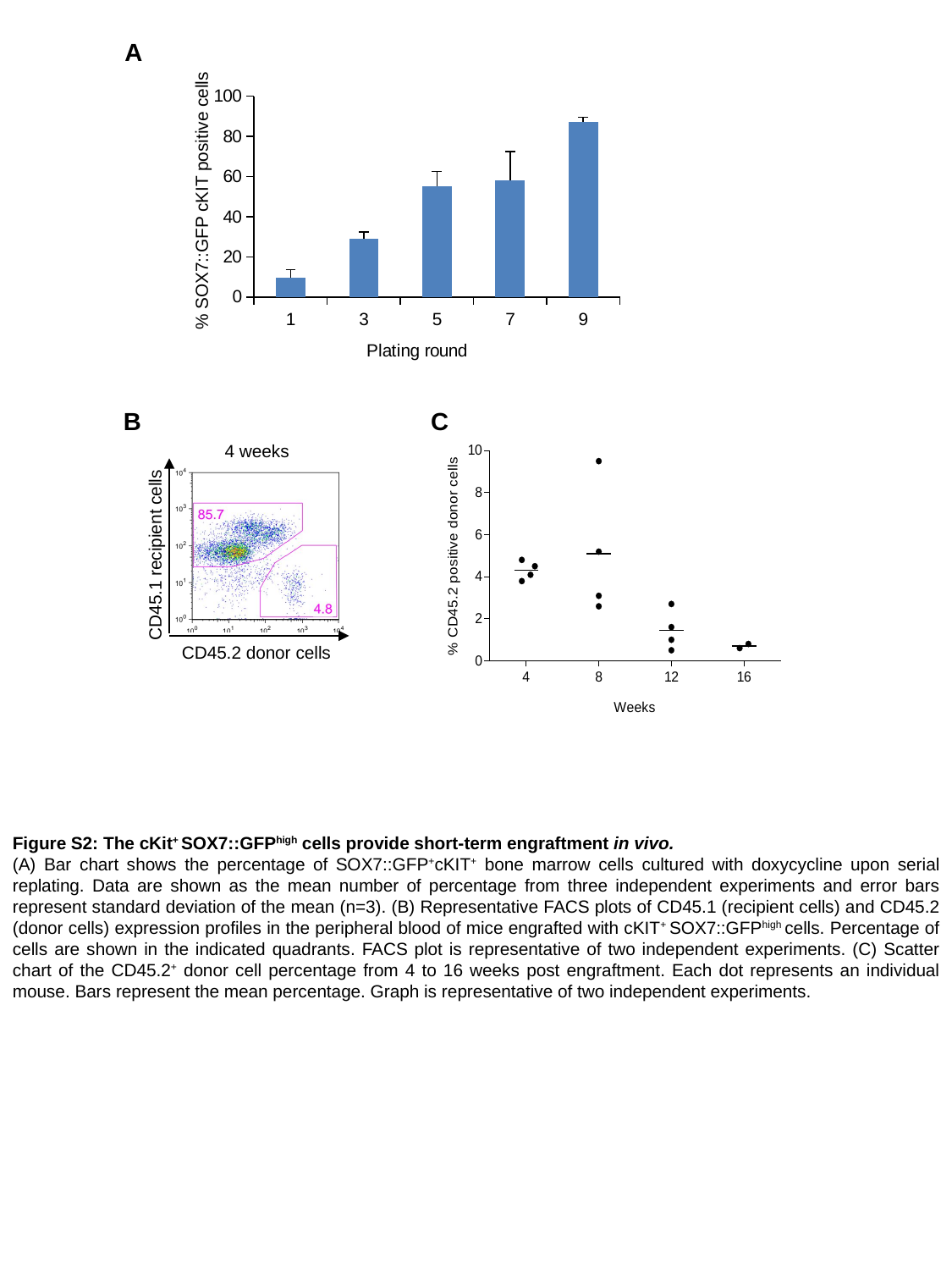

A
### Chart
| Category | |
|---|---|
| 1 | 9.45666666666667 |
| 3 | 29.2 |
| 5 | 55.26666666666645 |
| 7 | 57.95 |
| 9 | 87.4 |% SOX7::GFP cKIT positive cells
C
B
4 weeks
CD45.1 recipient cells
CD45.2 donor cells
Figure S2: The cKit+ SOX7::GFPhigh cells provide short-term engraftment in vivo.
(A) Bar chart shows the percentage of SOX7::GFP+cKIT+ bone marrow cells cultured with doxycycline upon serial replating. Data are shown as the mean number of percentage from three independent experiments and error bars represent standard deviation of the mean (n=3). (B) Representative FACS plots of CD45.1 (recipient cells) and CD45.2 (donor cells) expression profiles in the peripheral blood of mice engrafted with cKIT+ SOX7::GFPhigh cells. Percentage of cells are shown in the indicated quadrants. FACS plot is representative of two independent experiments. (C) Scatter chart of the CD45.2+ donor cell percentage from 4 to 16 weeks post engraftment. Each dot represents an individual mouse. Bars represent the mean percentage. Graph is representative of two independent experiments.

## Slide 3
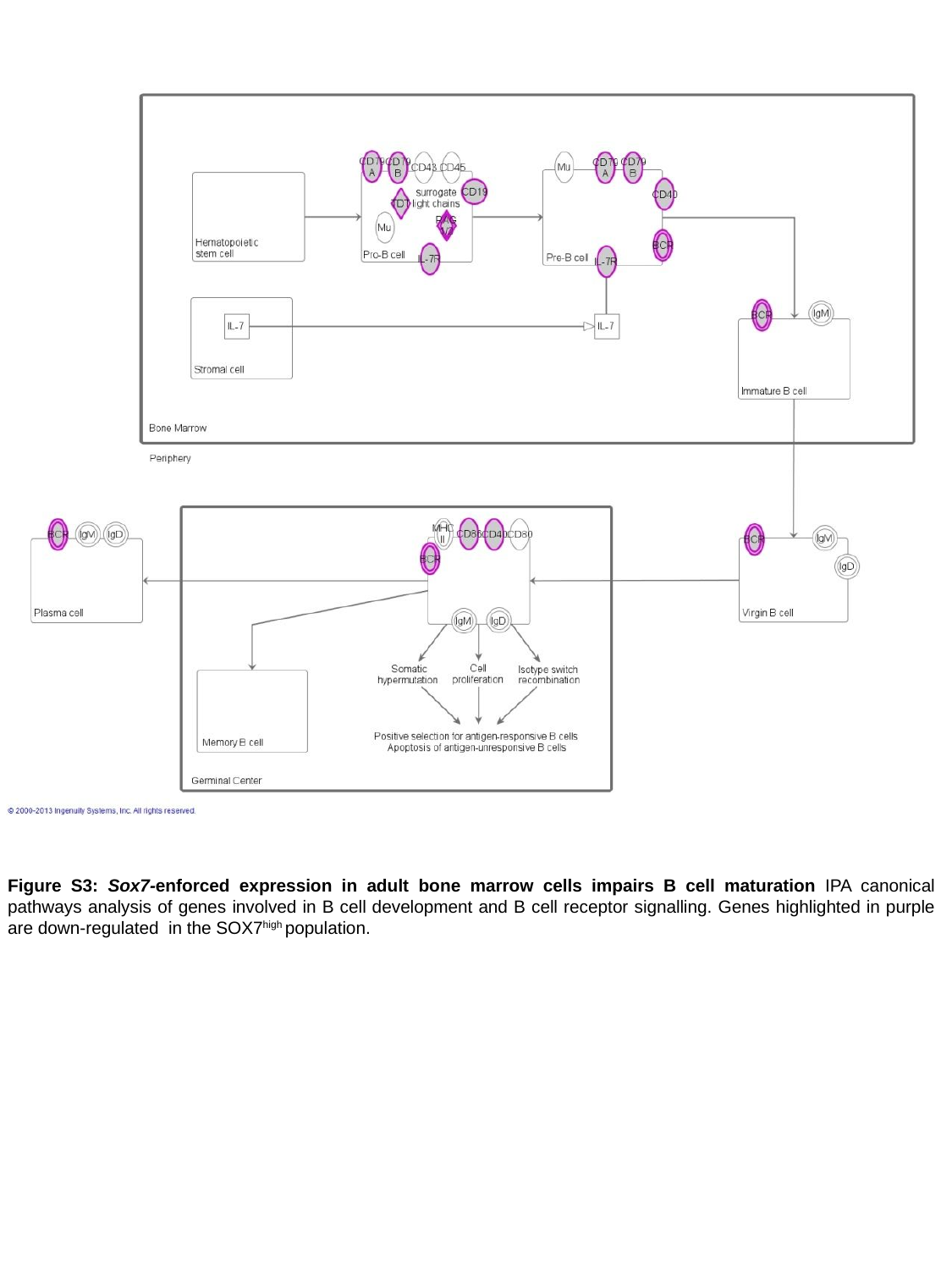

Figure S3: Sox7-enforced expression in adult bone marrow cells impairs B cell maturation IPA canonical pathways analysis of genes involved in B cell development and B cell receptor signalling. Genes highlighted in purple are down-regulated in the SOX7high population.

## Slide 4
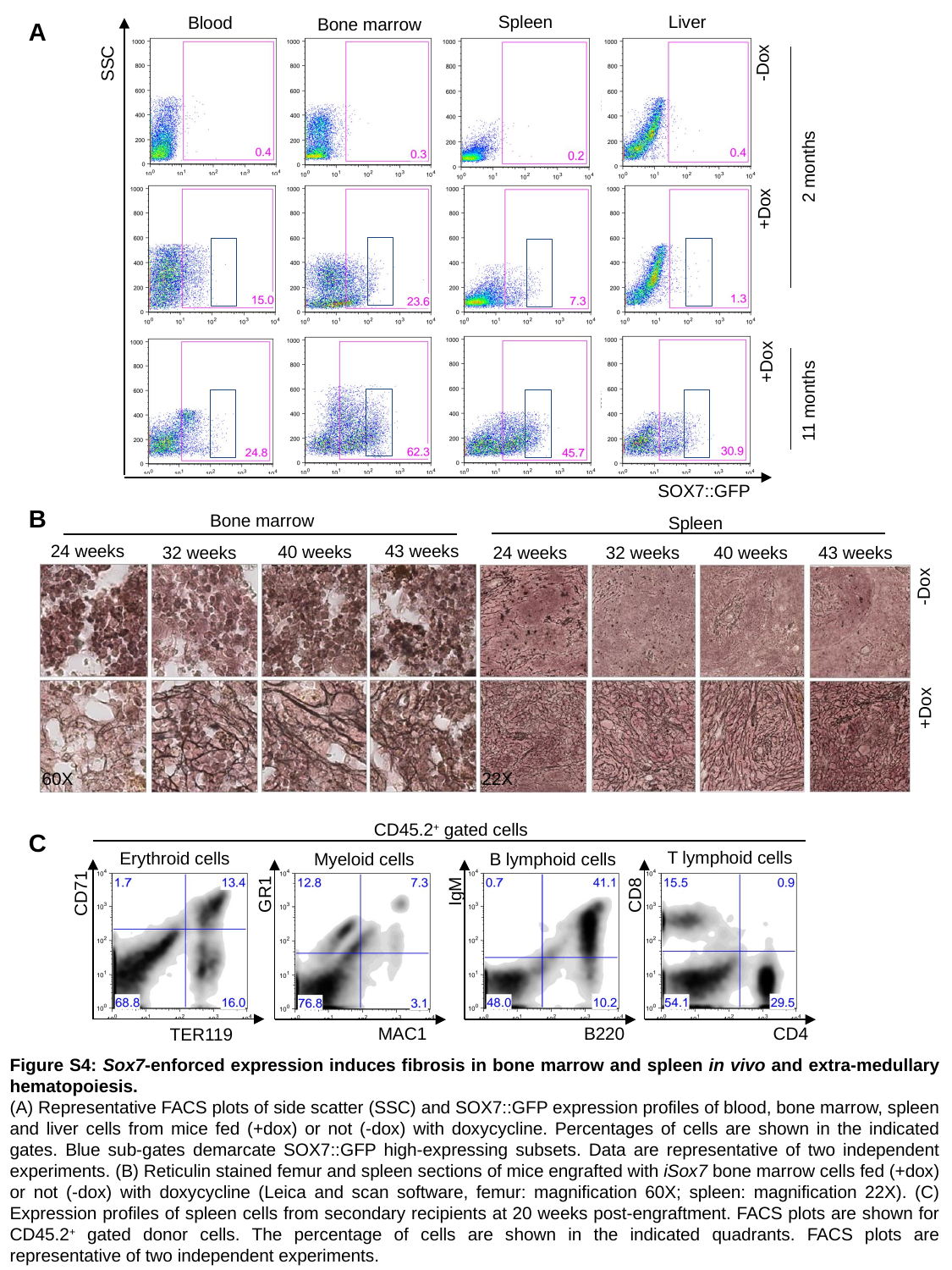

Spleen
Liver
Blood
Bone marrow
-Dox
SSC
2 months
+Dox
+Dox
11 months
SOX7::GFP
A
B
Bone marrow
Spleen
24 weeks
43 weeks
40 weeks
24 weeks
32 weeks
32 weeks
40 weeks
43 weeks
-Dox
+Dox
60X
22X
CD45.2+ gated cells
C
T lymphoid cells
Erythroid cells
B lymphoid cells
Myeloid cells
CD8
GR1
IgM
CD71
MAC1
B220
CD4
TER119
Figure S4: Sox7-enforced expression induces fibrosis in bone marrow and spleen in vivo and extra-medullary hematopoiesis.
(A) Representative FACS plots of side scatter (SSC) and SOX7::GFP expression profiles of blood, bone marrow, spleen and liver cells from mice fed (+dox) or not (-dox) with doxycycline. Percentages of cells are shown in the indicated gates. Blue sub-gates demarcate SOX7::GFP high-expressing subsets. Data are representative of two independent experiments. (B) Reticulin stained femur and spleen sections of mice engrafted with iSox7 bone marrow cells fed (+dox) or not (-dox) with doxycycline (Leica and scan software, femur: magnification 60X; spleen: magnification 22X). (C) Expression profiles of spleen cells from secondary recipients at 20 weeks post-engraftment. FACS plots are shown for CD45.2+ gated donor cells. The percentage of cells are shown in the indicated quadrants. FACS plots are representative of two independent experiments.
